# Association Between Patient Activation and Medication Adherence in Patients With Stroke: A Cross-Sectional Study

**DOI:** 10.3389/fneur.2021.722711

**Published:** 2021-09-30

**Authors:** Weijing Sui, Li-hong Wan

**Affiliations:** ^1^Nursing Department, Sir Run Run Shaw Hospital, Zhejiang University School of Medicine, Hangzhou, China; ^2^School of Nursing, Sun Yat-sen University, Guangzhou, China

**Keywords:** stroke, patient activation, medication adherence, cross-sectional study, secondary prevention

## Abstract

**Background:** Medication adherence is key to secondary prevention in patients with stroke. Poor medication adherence can lead to recurrence, disability, or even death in stroke survivors. Patient activation is associated with increased healthy behaviors and improved clinical outcomes in many chronic diseases. However, the association between patient activation and medication adherence in patients with stroke remains unclear.

**Objective:** The study aimed to explore the influence of patient activation on the medication adherence of patients with stroke and to analyze the reasons for medication nonadherence.

**Materials and Methods:** A cross-sectional design with convenience sampling was used in this study. A total of 119 patients with stroke were recruited from a tertiary hospital in Guangzhou. A social-demographic and clinical data form, a self-developed medication adherence questionnaire, and the 13-item Patient Activation Measure (PAM-13) were used. Univariate analysis and multiple linear regression analysis with dummy variables were conducted to investigate the associations between medication adherence and patient activation. Data were analyzed with IBM® SPSS® version 25.0.

**Results:** The mean PAM-13 score in patients with stroke was 51.56 ± 12.58. A low level of patient activation was reported by up to 66.4% of the patients. The self-reported medication adherence questionnaire score was 5.59 ± 1.52. A low level of medication adherence was reported by up to 59.7% of the patients, while a moderate level was reported by 34.4%, and a high level was reported by only 5.9%. In the multiple stepwise regression analysis, patient activation was found to be an independent influencing factor of medication adherence in patients with stroke (*p* < 0.05).

**Conclusion:** Medication adherence was poor in patients in Guangzhou, China, following an ischemic stroke. Patient activation as the independent influencing factor identified in this study will support healthcare givers to develop the tailored intervention to improve medication adherence among patients with stroke in China.

## Introduction

Stroke is characterized by a sudden onset and rapid occurrence of localized or diffuse brain function defects. It is a group of common cerebrovascular diseases caused by organic brain injury. Stroke includes transient ischemic attack (TIA), ischemic stroke, and hemorrhagic stroke (1). Ischemic stroke is the most common type of stroke, accounting for almost 80% of all strokes (2).

Stroke has become a major public health problem; it is harmful to human health and has high mortality and disability worldwide, affecting millions of people every year (3). Approximately 90% of stroke survivors have varying degrees of functional impairment (4). In low- and middle-income countries, the stroke burden is particularly heavy (5, 6). China is no different. With the acceleration of social aging and urbanization, the popularity of unhealthy lifestyles, and widespread exposure to cerebrovascular risk factors, the stroke burden in China has shown an explosive growth trend (7). The standardized incidence of the first stroke among residents 40–74 years old in China has increased by an annual rate of 8.3% (7). Therefore, the prevention and treatment of stroke face enormous challenges (7).

Post-stroke treatment mainly relies on long-term repair strategies, such as drug therapy, physical exercise, and a healthy lifestyle (8). Among them, drug therapy is the most important component in the medical treatment of stroke (8). Studies on the etiology and pathogenesis of stroke (9) have shown that long-term drug treatment can effectively control blood pressure, improve cerebral blood circulation, prevent clogged distal small blood vessels from secondary thrombosis, and reduce the generation of new microembolisms, thereby reducing the recurrence or fatality rates of stroke (10). The types of medication used in patients with stroke usually include antihypertensive drugs, antiplatelet aggregation drugs, and lipid-lowering drugs, as well as other drugs needed for the treatment of coexisting diseases (10). Medication adherence commonly refers to the behavior in which patients take their medicine according to the doctor's advice and emphasizes the patients' behavior of receiving certain drug treatments and the participation, compliance, and persistence of maintaining a certain drug treatment program (11). For stroke patients, the ability to follow the doctor's advice to take medications correctly and for a long time is the fundamental prerequisite to ensure the effectiveness of drug treatment.

However, poor medication adherence is considered to be the biggest problem at present. The WHO noted that “drug non-compliance is a major problem around the world”. Among patients with chronic illness, approximately 33–50% of patients do not adhere to a long-term medication regimen (10). The medication compliance of apoplectic patients is generally poor (12). A systematic review showed that nearly one-third of patients do not comply with medication plans (13). With the increasing incidence of stroke, the phenomenon of poor drug compliance may be aggravated in China. The reasons why patients do not follow their medication plan are complex. Many factors are related to medication compliance in patients with stroke, such as sociodemographic factors, clinical factors, disease perception, and beliefs about medication (14). Cognitive impairment and the accompanying emotional changes, such as depression or fatigue, may put patients with stroke at greater risk for poor medication adherence (15). In addition, poor adherence to stroke medications is also related to the low utilization rate of health resources (14, 16).

Considering these complex and vexing situations, active patient involvement in treatment is very important to improve the effectiveness of treatment and control stroke and its sequelae. Therefore, Dr. Hibbard proposed the concept of patient activation based on the theories of self-efficacy, self-management, and self-discipline (14, 16). Patient activation means that patients recognize the important role they play in the process of self-management, health maintenance, and communication and collaboration with healthcare providers (17). That is, patients have the responsibility, confidence, knowledge, and skills to manage their health (17). Individuals who are more activated are more likely to make rational decisions and carry out prescribed activities consistent with those of stroke treatment plans (18). Substantial evidence supports patient activation as a reliable influencing factor related to better clinical outcomes and healthier behaviors in chronic diseases (19, 20).

Adhering to medications plays a pivotal role in secondary prevention in patients with stroke. However, medication compliance in patients with stroke is generally poor (12). The factors influencing drug compliance are numerous and complex; among them, patient activation is supposed to be associated with healthy behaviors. Therefore, research on the association between patient activation and medication adherence should be strengthened to effectively improve drug compliance in patients with stroke who need long-term treatment (21). There is a paucity of known studies on the relationship between patient activation and medication adherence among patients with stroke, especially in Chinese patients. This study aims to explore the relationship between patient activation and medication adherence in patients with stroke, then to provide a reference for healthcare providers to formulate targeted intervention measures, thereby improving clinical outcomes.

## Materials and Methods

### Theoretical Framework

The chronic disease care model (CCM) was put forward by Wagner of the MacColl Health Care Innovation and Development Association in 1998 (22). It is an evidence-based, systematic, and comprehensive care model based on the summary of many chronic disease care practices, and it is used to explain the process of formation of chronic disease care outcomes to guide chronic disease care interventions and increase output. The CCM includes eight elements: (i) Community Resources and Policies; (ii) Health Care Organization; (iii) Self-management Support; (iv) Delivery System Design; (v) Decision Support; (vi) Clinical Information Systems; (vii) Prepared, Proactive Practice Team; and (viii) Informed, Activated Patient. Based on the first six elements, through fully informed and activated patients, interactive communication with well-prepared medical teams forms the clinical and behavioral outcome of chronic disease. Under the framework of the CCM, Hibbard et al. (17) proposed the concept of patient activation, pointing out that patients should play an active role in the healthcare system. The higher the level of patient activation is, the easier it is for patients to adopt healthy behaviors (23).

**Figure 1:**
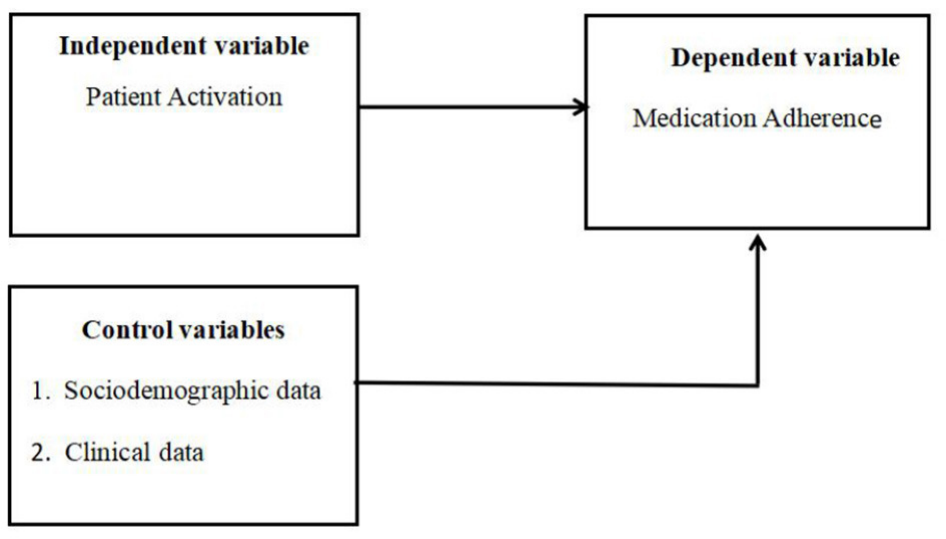
The theoretical framework of this study.

The theoretical framework of this study is based on the purpose of the study (As shown in Figure 1). In this study, the dependent variable was medication adherence. The independent variable was patient activation. The other variables that may affect the dependent variable are the control variables, which included sociodemographic data (age, sex, education, employment status, marital status, place of residence, family per capita monthly income, medical payment method, etc.) and clinical data [BMI index, the type of stroke, course of stroke (years), initial stroke or not, the type and number of coexisting diseases, disturbance of limb activity, activities of daily living, and medication regimen]. The theoretical framework of this study is as follows:

### Study Design and Participants

This study adopted a cross-sectional correlational method with convenience sampling. Moreover, a formal sample size calculation was not done. The study included participants who were (i) clinically diagnosed with ischemic stroke confirmed by CT or MRI; (ii) stroke outpatients who had taken long-term medication at home for 1 month or more after clinical diagnosis; (iii) conscious and able to communicate in Cantonese or Mandarin, (iv) at least 18 years of age or older, and (v) signed an informed consent form. The study excluded participants who (i) had sensory aphasia or total aphasia and were unable to participate in basic communication; (ii) were considered to have dementia by the Mini-Mental State Examination (MMSE) (24) (education level in primary school and below ≤ 17 points, in junior high school, and above ≤ 22 points) or mental disorders; or (iii) also had severe heart, liver, kidney and lung insufficiency, malignant tumor, etc.

### Study Instruments

#### Sociodemographic Form

A sociodemographic form was used to record the sociodemographic data of patients with stroke, including age, sex, education, occupational status, marital status, place of residence, living alone or not, family per capita monthly income, and medical payment methods. The sociodemographic data form was completed by patients independently.

#### Clinical Data Form

Clinical data included body mass index (BMI) (25), medical diagnosis, initial stroke or not, the type and number of coexisting diseases, time from stroke (in years), activities of daily living (ADL) level, limb movement disorder, and drug treatment. BMI was grouped according to Chinese adult standards: 18.5 ≤ BMI <24 is normal, 24 ≤ BMI <28 is overweight, and BMI ≥ 28 is obesity. The ADL level of patients with stroke was evaluated by the Barthel Index (26), which has good reliability and validity. The scale includes 10 items such as defecation control, grooming, toilet use, eating, bed-to-chair transfer, activities (walking in and around the room, excluding long walks), going up and down the stairs (independent of a walking stick), and taking a bath. The Barthel Index score ranges from 0 to 100 with 100 corresponding to complete self-care in activities of daily life, 60–99 corresponding to mild dysfunction, 40–59 corresponding to moderate dysfunction, 20–39 corresponding to severe dysfunction, and 0–19 corresponding to extremely severe dysfunction (26). The five-level myodynamia was used to determine whether the patient has limb movement disorder: level 0 means completely paralyzed, and no muscle contraction can be measured; level 1 means only muscle contraction is measured, but no movement can be produced; level 2 means limbs can move in parallel on the bed, but cannot resist their gravity, that is, they cannot lift off the bed; level 3 means limbs can overcome gravity and lift off the bed surface, but cannot resist resistance; level 4 means the limbs can do exercises against external resistance, but not completely; and level 5 means muscle force is normal. In this study, the myodynamia level 0–3 means that the patient has a limb movement disorder, while the 4–5 level means that there is not. The clinical data form was completed by the researchers by consulting the electronic medical record.

#### The 13-Item Patient Activation Measure

The Patient Activation Measure (PAM) was originally developed by Hibbard (17) using a 22-item scale, which was later reduced to 13 items. It is an interval level, Guttman-like scale designed to provide an objective characterization of an individual's activation level on a developmental spectrum of knowledge and capacity to take on the role of self-management (17). The four dimensions of the PAM-13 correspond to four patient activation levels: believes active role as important (level 1); confidence and knowledge to take action (level 2); taking action (level 3); and staying the course under stress (level 4) (17, 27). The Chinese version of the PAM-13 was used in this study. This was authorized by Insignia Health, LLC 2011 (OR, USA). The measure uses a five-point Likert response scale. The higher the score, the better the activation level. The Chinese version of the PAM-13 has shown good reliability and validity, with a Cronbach's α of 0.920 (28).

#### Self-Reported Medication Adherence Questionnaire

Consensus on a gold-standard measure of patient medication nonadherence has been elusive, in part because medication nonadherence involves multiple, distinct behaviors across three phases (initiation, implementation, and persistence). To assess these behaviors, the consensus was achieved for eight patient medication nonadherence behaviors determined through literature review (29) and two rounds of the Delphi method: not filling an initial prescription, not taking the first dose, refilling prescription late, missing doses, taking extra doses, taking doses at the wrong time, improperly administering medication, and discontinuing medication early. Adapted to the needs of the current study, stroke medication adherence was measured by a self-developed medication adherence questionnaire, which was developed based on related literature and publicly available scales (30–32). It was a structured self-reported measurement of drug use behavior. The questionnaire was designed to help identify disorders and behaviors related to long-term medication adherence. This measure provides information on behaviors, both unintentional and intentional, related to medication use. The questions were formulated to avoid “yes” bias. The answers to the first seven items are divided into two categories, “Yes” or “No” with 1 for “No” and 0 for “Yes”; items 5 and 8 are reverse scored. The answer to item 8 uses a five-point Likert response scale, which is divided into never, rarely, sometimes, often, and always, and the corresponding scores are 1, 0.75, 0.5, 0.25, and 0, respectively. The total medication adherence questionnaire score ranged from 0 to 8. The higher the score, the better the medication adherence: <6 was considered low adherence, 6–8 (excluding eight points) was considered medium adherence, 8 points were considered high adherence. Evidence of good reliability and validity of the self-reported medication adherence questionnaire has been provided.

### Statistical Analysis

After all the data were collected, the database was established. The questionnaire data were entered into the database with double data entry. IBM SPSS 25.0 was used for statistical analysis.

Medication adherence was taken as the dependent variable. The independent variables were mainly some factors that were statistically significant in univariate analysis [age, educational level, initial stroke or not, time from stroke (in years), the number of coexisting diseases] and some significant independent factors in the literature review (patient activation) (19–21). Among them, the unordered multiple categorical variables and ordered multiple categorical variables were converted into multiple dichotomous “dummy variables.” There were a total of 17 variables including dummy variables in this study. According to the requirements of the logistic regression model, the sample size should be 5–10 times the number of independent variables (33, 34). The calculation results showed that it would take at least 85 (85–170 range) patients to achieve a power of 0.80, with a two-tailed α of 0.05. Considering a loss rate of 10–20%, we should enroll about 94–102 patients. Among the questionnaires answered by the included patients, more than 50% of the vacant items will be eliminated. A two-sided test was used for statistical inference, and the significance level was α = 0.05. Frequency, percentage, mean, and standard deviation were used to describe the general characteristics of the included patients with stroke. Frequency, percentage, median, mean, and standard deviation were used to describe patient activation and medication adherence status.

Univariate analysis was conducted by corrected *t*-test or analysis of variance to explore the effect of sociodemographic and clinical data on medication adherence in patients with stroke. The dummy variables were analyzed by the Enter regression method, and the other binary variables were analyzed by the stepwise regression method in multiple linear regression analyses. In the analysis, we drew a histogram of the residuals to test the normality, drew a scatter plot of the residuals vs. the predicted value of the reaction variable to test the linearity and homogeneity of the variance, and calculated the Durbin–Watson statistics for independent tests. To reasonably estimate and interpret the regression model, we determined the factors with statistical significance (*p* < 0.05) by univariate analysis. The variance inflation factor (VIF), combined with professional knowledge, was used to judge whether there was collinearity; after variable elimination, the VIF of the final selected variables were all <3, which was considered statistical evidence that there was no collinearity between the variables. Multiple linear regression analysis with stepwise model selection was carried out to determine the factors influencing medication adherence in stroke patients, taking medication adherence as the dependent variable and sociodemographic data, clinical data, and patient activation as the independent variables.

### Ethical Considerations

Ethical approval was obtained from the Sun Yat-sen University School of Nursing and the third affiliated hospital. Written consent was obtained from all participants. In this study, an anonymous survey was used to obtain the patients' informed consent without deception or reluctance. During the study, patients had the autonomy to decide to suspend their participation or withdraw from the study. The information collected with this survey is only used for research, strictly confidential, and never leaked. The measurements used in this study were all approved by the developers before implementation.

## Results

### Participant Characteristics

From July 2015 to October 2015, 127 patients who met the study criteria were included. When we collected the questionnaires, we found that the vacant items of eight questionnaires from patients exceeded 50%, so we eliminated them. A total of 119 patients fully completed the measurement for a response rate of 93.70%.

The social-demographic and clinical data are shown in Table 1. The age of the participants ranged from 23 to 91, with a mean of 64.35 (SD = 11.96). The ratio of males (58.0%) to females (42.0%) was near unity. The majority of the participants were middle-aged or elderly patients (*n* = 105, 88.2%), married (*n* = 115, 96.6%), living with family (*n* = 114, 95.8%), unemployed or nonemployed (*n* = 97, 81.5%). More than half of the participants' educational level was a secondary school or an undergraduate degree (*n* = 75, 63.0%). A small minority (*n* = 18, 15.1%) were from low-income families (the per capita monthly income of the family <2,000 RMB). The proportion of stroke patients living in urban areas (50.4%) was almost the same as in the proportion living in rural areas (49.6%). More than half of the patients were overweight or obese (*n* = 61, 51.3%). In terms of the medical payment method, the proportion of out-of-pocket patients (54.0%) was almost the same as the proportion of patients with various health insurance (46.0%).

**Table 1:** Sociodemographic and clinical data of the participants (*n* = 119).

	**Frequency (*n*)**	**Percentage (%)**
**Sociodemographic variables**		
Sex		
Male	69	58.0
Female	50	42.0
Age (years)		
23~44	14	11.8
45~59	39	32.8
60~74	41	34.4
75~91	25	21.0
Family per capita monthly income (RMB)		
<2,000	18	15.1
2,000~2,999	29	24.4
3,000~3,999	28	13.5
4,000~4,999	20	16.8
≥5,000	24	20.2
BMI		
18.5 ≤ BMI <24	58	48.7
24 ≤ BMI <28	48	40.4
BMI ≥ 28	13	10.9
Housing situation		
In nursing home	1	0.8
Living alone	4	3.4
Living with family	114	95.8%
Place of residence		
Urban areas	60	50.4
Rural areas	59	49.6
Educational level		
Primary school or lower	44	37.0
Junior middle school	21	17.6
Senior high school	25	21.0
Bachelor's degree	29	24.4
Medical payment method		
Out of pocket	54	45.4
The new rural cooperative medical system	4	3.4
Urban medical insurance	51	42.8
Commercial health insurance	4	3.4
Government insurance	6	5.0
Employment status		
Employed	22	18.5
Retired	53	44.5
Unemployed or nonemployed	44	37.0
Marital status		
Unmarried	4	3.4
Married	115	96.6
**Clinical characteristic**		
Diagnosis on admission		
Transient ischemic attack	12	10.1
Ischemic stroke	107	89.9
Family history of stroke		
Yes	12	10.1
No	107	89.9
Barthel Index (points)		
20~	10	8.4
60~	22	18.5
100	87	73.1
Medication regimen		
Antihypertensive	93	78.2
Lipid-regulating drugs	94	78.9
Antiplatelet drug	89	74.8
Hypoglycemic drugs	37	31.1
Time from stroke (in years)		
<1	66	55.5
1–2	22	18.5
3–4	20	16.8
≥5	11	9.2
Initial stroke		
Yes	57	47.9
No	62	52.1
Limb movement disorder		
Yes	32	26.9
No	87	73.1
The number of coexisting diseases		
0	8	6.7
1	51	42.9
≥2	60	50.4
Coexisting diseases		
Hypertension	93	78.2
Diabetes	37	31.1
Dyslipidemia	94	78.9
Previous myocardial infarction	1	0.8
History of atrial fibrillation	2	1.6
Coronary heart disease	11	8.8
Peripheral arterial disease	1	0.8

In terms of clinical characteristics, the vast majority had cerebral infarction (*n* = 107, 89.9%). A minority of patients had a family history of stroke (*n* = 12, 10.1%). The proportion of patients with recurrent stroke (52.1%) was almost the same as that of first-episode patients (47.9%). Nearly half of the patients had a stroke <1 year prior (*n* = 66, 55.5%). The vast majority of stroke patients had one or more coexisting diseases, including hypertension, diabetes, hyperlipidemia, peripheral artery disease, atrial fibrillation, and previous myocardial infarction. In the medication regimens, most patients used antihypertensives (*n* = 93, 78.2%), lipid-regulating drugs (*n* = 94, 78.9%), and antiplatelet drugs (*n* = 89, 74.8%), and a small number of patients use hypoglycemic drugs (*n* = 37, 31.1%). A small number of patients had impaired limb movement disorder (*n* = 32, 26.9%). Most patients had normal ADL ability and were able to take care of themselves (*n* = 109, 91.6%).

### Patient Activation Status

In this study, the Cronbach's α of the PAM-13 was 0.823, showing that it had good internal consistency and reliability and can be used to effectively measure the patient activation level.

The raw patient activation scores of the 119 patients with stroke in this study were converted to standard scores and are presented in Table 2; the score range was 35.5–100. The median was 47. The mean plus or minus the standard deviation was 51.56 ± 12.58, which was equivalent to patient activation level 2. We can conclude that the overall level of stroke patient activation was extremely low. In addition, more than half of the stroke patients (*n* = 79, 66.4%) were at a low level (levels 1 and level 2).

**Table 2:** The patient activation level in patients with stroke (*n* = 119).

**The level of patient activation**	**Frequency *(n)***	**Percentage (%)**	**Mean (**$\bar{x}$ **±*s*)**
Level 1 (<47)	63	52.9	42.72 ± 3.00
Level 2 (47.1–55.1)	16	13.5	50.90 ± 1.83
Level 3 (55.2–67)	26	21.8	58.95 ± 3.58
Level 4(>67.1)	14	11.8	78.36 ± 10.16
Total	119	100.0	51.56 ± 12.58

### Medication Adherence Status

In our study, the Cronbach's α of the self-reported medication adherence questionnaire was 0.746, showing that it had good internal consistency and can be used to measure the medication adherence behavior reliably. The average medication adherence score of the 119 stroke patients included in this study was 5.59 ±1.52 (a low level), as shown in Table 3. Among the participants, ~5.9% (*n* = 7) had a high level of medication adherence. Approximately 34.4% (*n* = 41) were at a medium level. Approximately 59.7% (*n* = 71) were at a low level. According to the further analysis of each item, the compliance rate was the highest for “Did you take your medication correctly yesterday?”, which suggests that most stroke patients can follow the doctor's advice to take their medicine correctly for a short period time. The compliance rate was the lowest for “How often did you ever have difficulty remembering to take your medication?”, which suggests that there are some barrier factors in the behavior of taking medicine in compliance with the doctor's instructions.

**Table 3:** The self-reported medication adherence questionnaire scores in patients with stroke (*n* = 119).

**Item**	**Adherence (*n*)**	**Adherence Rate (%)**	**Mean (**$\bar{x}$ **±*s)***
Did you take your medication as prescribed yesterday?	115	96.6	0.96 ± 0.60
Over the past seven days, were there any days when you did not take medication?	103	86.6	0.87 ± 0.34
Do you ever forget to take your medicine?	96	80.7	0.81 ± 0.40
When you are not at home, do you take all your medications as prescribed?	82	68.9	0.69 ± 0.47
Have you ever cut back on or stopped taking the medication without telling the doctor?	72	60.5	0.61 ± 0.49
When you feel like your stroke is under control, do you sometimes reduce your dosage or withdraw the medication?	70	58.8	0.59 ± 0.50
Do you ever feel hassled about adhering to your medication treatment plan?	50	42.0	0.42 ± 0.50
How often do you feel you have difficulty remembering to take medication?	22	18.5	0.68 ± 0.22
The Total	7	5.9	5.59 ± 1.52

### Association Between Patient Activation and Medication Adherence

#### Univariate Analysis of Medication Adherence

As shown in Table 4, univariate analysis showed that there were significant differences in medication adherence among patients of different ages, employment status, place of residence, education level, and family per capita monthly income (*p* < 0.05). The compliance of patients aged 45–75 years was better than that of patients over 75 years old. The medication compliance of retired patients was better than that of employed patients. The medication compliance of stroke patients in urban areas was better than that of patients in rural areas. The medication compliance of patients with an education level of senior high school or bachelor's degree was better than that of patients with an education level of primary school or below. The compliance of patients with a per capita monthly income of >4,000 RMB was better than that of those with a per capita monthly income of <2,000 RMB. Regarding clinical characteristics, there were significant differences in medication adherence according to whether this was their initial stroke, time from stroke (in years), and the number of coexisting diseases (*p* < 0.05). The medication compliance of recurrent patients was better than that of initial stroke patients. The medication compliance of stroke patients with a course of >5 years was better than that of patients with a course of 3–5 years or <1 year. Stroke patients without coexisting diseases or with two or more coexisting diseases had better drug compliance than patients with one coexisting disease.

**Table 4:** Univariate analysis of the influence of sociodemographic and clinical data on medication adherence (*n* = 119, $\bar{x}$ ±*s*).

	**Frequency *(n)***	**Medication adherence**	***t*/*F***	***p***
Sex			1.666	0.098
Male	69	5.79 ± 1.56		
Female	50	5.32 ± 1.44		
Age (years)			17.034	0.000^*^
23~44**①**	14	4.66 ± 0.99		**③** > ①^*^
45~59**②**	39	5.71 ± 1.59		**②** > ④^*^
60~74**③**	41	6.52 ± 1.22		**③** > ④^*^
75~91**④**	25	4.39 ± 0.93		
Employment status			4.513	0.013^*^
Employed**①**	22	5.08 ± 1.64		**②** > ①^*^
Retired**②**	53	6.05 ± 1.70		
Unemployed **③**	44	5.67 ± 1.57		
Medical payment method			1.716	0.151
Out of pocket	54	5.27 ± 1.82		
The new rural cooperative medical system	4	4.75 ± 1.71		
Urban medical insurance	51	5.97 ± 1.51		
Commercial health insurance	4	6.18 ± 1.04		
Government insurance	6	6.13 ± 1.91		
BMI			0.150	0.861
18.5 ≤ BMI <24	58	5.68 ± 1.80		
24 ≤ BMI <28	48	5.65 ± 1.50		
BMI ≥ 28	13	5.29 ± 1.99		
Place of residence			12.741	0.001^*^
Urban areas	60	6.17 ± 1.55		
Rural areas	59	5.07 ± 1.67		
Education level			9.508	0.000^*^
Primary school **①**	44	4.75 ± 1.48		**③** > ①^*^
Junior middle school **②**	21	5.58 ± 1.50		**④** > ①^*^
Senior high school **③**	25	5.97 ± 1.87		**④** > ②^*^
Bachelor degree **④**	29	6.56 ± 1.30		
Marital status			0.881	0.453
Unmarried	4	6.50 ± 1.06		
Married	115	5.59 ± 1.72		
Family per capita monthly income (RMB)			6.884	0.000^*^
<2,000 **①**	18	4.45 ± 1.71		**④** > ①^*^
2,000~2,999 **①**	29	4.98 ± 1.40		**④** > ②^*^
3,000~3,999 **③**	28	5.67 ± 1.53		**⑤** > ①^*^
4,000~4,999 **④**	20	6.90 ± 1.15		
≥5,000 **⑤**	24	6.17 ± 1.76		
Living alone			3.425	0.312
Yes	4	6.00 ± 0.67		
No	115	5.85 ± 1.54		
Initial stroke			19.598	0.000^*^
Yes	62	4.98 ± 1.75		
No	57	6.33 ± 1.34		
Limb movement disorder			1.751	0.188
Yes	34	5.84 ± 1.64		
No	85	5.09 ± 1.75		
Time from stroke(in years)			2.759	0.045^*^
<1 **①**	66	5.34 ± 1.80		**④** > ①^*^
1–2**②**	22	6.17 ± 1.34		
3–4**④**	20	5.62 ± 1.61		
≥5 **⑤**	11	6.27 ± 1.51		
Barthel Index			0.027	0.973
20~	10	5.27 ± 1.78		
60~	22	5.43 ± 1.70		
100	87	5.72 ± 1.70		
Family history of stroke			1.398	0.239
Yes	12	5.63 ± 1.68		
No	107	5.62 ± 1.94		
The number of coexisting diseases			7.737	0.000^*^
0 **①**	8	6.08 ± 1.70		**①** > ②^*^
1 **②**	51	4.99 ± 1.32		**③** > ②^*^
≥2 **③**	60	6.02 ± 1.50		

* *p < 0.05*.

#### Correlation Between Patient Activation and Medication Adherence

Spearman correlation analysis was used to analyze the correlation between patient activation and medication adherence. As shown in Table 5, the total PAM-13 score was positively correlated with the self-developed medication adherence questionnaire score (*r* = 0.496, *p* < 0.05). The four dimensions of the PAM-13 (believes active role as important, confidence and knowledge to take action, taking action, and staying the course under stress) were positively correlated with the self-developed medication adherence questionnaire score (*r*_1__=_ 0.457, *r*_2__=_ 0.406, *r*_3__=_ 0.416, *r*_4__=_ 0.388, *p* < 0.05).

**Table 5:** Correlation between patient activation and medication adherence in patients with stroke (*n* = 119).

		**Patient activation**			
	**Total score**	**Believes active role important**	**Confidence and knowledge to take action**	**Taking action**	**Staying the course under stress**
**Medication adherence**	0.496^*^	0.457^*^	0.406^*^	0.416^*^	0.388^*^

* *p < 0.05*.

#### Multivariate Analysis of Medication Adherence

Medication adherence was taken as the dependent variable. The items with statistical significance in univariate analysis [age, educational level, initial stroke, time from stroke (in years), the number of coexisting diseases, and patient activation] were included in multiple linear regression analysis as independent variables. The results of the multivariate analysis showed that three factors, patient activation, recurrent stroke, and educational status, remained in the linear regression equation with medication adherence as the dependent variable. The regression equation, Ŷ = 2.747 + 0.04$0^{X_{1}}$ + 0.77$4^{X_{2}}$ + 0.76$4^{X_{3}}$ + 0.90$9^{X_{4}}$, *R*^2^= 0.365, explained ~36.5% of the stroke patients' medication adherence. Among the independent variables, the influence of patient activation, recurrent stroke, and educational level on medication adherence was positive (Table 6).

**Table 6:** Multivariate analysis of medication adherence (*n* = 119).

**Variables**	**Partial regression coefficient**	**The standardized partial regression coefficient**	***t***	***p***
Constant	2.747		5.596	0.000^*^
^*X*__1_^ patient activation	0.040	0.328	3.997	0.000^*^
^*X*__2_^ recurrent stroke	0.774	0.255	3.279	0.001^*^
^*X*__3_^ education level (senior high school)	0.764	0.205	2.358	0.020^*^
^*X*__4_^ education level (bachelor's degree)	0.909	0.257	2.788	0.006^*^

*R = 0.605, R^2^= 0.365, $R_{\text{ad}}^{2}=$ 0.337*;

* *p < 0.05*.

## Discussion

The first finding of this study was that medication adherence among patients with stroke in Guangzhou, China was generally poor, with over 90% of the participants having a medium to low level of compliance. This was in line with results reported from other studies (13, 21). Among the reasons indicated by the patients, the main problem affecting their medication adherence was having difficulty remembering to take all the medication. Most patients with stroke are treated with a combination of drugs. The number of drugs taken is numerous and complicated, and patients are required to adhere to the standard treatment for a long time, so it is difficult for patients to manage the use of these drugs (35). Current evidence has taken into account the role of biopsychosocial factors in trying to understand medication compliance. Influencing factors, such as increased concerns about prescription medications, low awareness of the benefits of medications, reduced cognitive function, and the presence of many coexisting diseases, have previously been identified among stroke survivors (12, 21, 36–38). Drug-related side effects and interactions, difficulty in obtaining prescriptions from doctors or pharmacies, and prescription cost issues may also lead to drug noncompliance (13, 39, 40). One study reported that medication adherence was significantly lower in stroke patients than in patients with other chronic diseases (35). This may be because the stroke victim's neurological symptoms are not immediately relieved markedly after taking medication. As a result, survivors of stroke do not feel the need to adhere to long-term medication. Therefore, the most important task for medical staff is to resolve the misunderstanding about the use of prescription drugs for stroke patients. Drug treatment is mainly used for secondary prevention, not for relieving neurological symptoms (10). Stroke patients with low educational levels have little knowledge of stroke prevention and control, so they tend to make irrational decisions and stop taking their medications.

Our study also shows that the activation level of stroke patients is extremely low. Patient activation refers to an individual's ability and willingness to assume the role of managing their health and wellness (17). Positive changes in patient activation can lead to changes in medication compliance of patients with chronic diseases (18, 41). In our study, a majority of patients were at level 1 and level 2. In the field of chronic diseases, the activation level of stroke patients reported in our study is lower than those reported by Macabe et al. (42) in the United States and Bos tou-wen (43) in the Netherlands. It may be that China is still in the stage of a developing country. The health literacy and healthcare awareness of the people need to be improved. Most stroke patients belong to the middle-aged and elderly groups. Under the long-time influence of the traditional medical treatment mode in China, they have the misconception that treatment is a matter for doctors and nurses, and whether the condition can be controlled and whether the stroke can relapse is only determined by the diagnosis and treatment level by medical staff. The patients have nothing to do with it. They do not realize that they must play a proactive role in self-care, which is an important factor in determining their health. They are prone to overreliance on the guidance and judgment of medical professionals, are unable to take action to manage their health, and cannot actively participate in the medical care team.

The independent influencing factors of drug adherence in Chinese stroke patients identified through this study are educational level and recurrent stroke. The results indicate that patients with stroke who have a higher educational level may have better medication adherence, which is consistent with other studies (21). The cause might be that the higher the educational level of patients is, the stronger their ability to obtain, use, and evaluate health-related information is (21). Patients with higher education levels can accept and understand the knowledge about medications and realize the serious consequences that may result from noncompliance (15). Therefore, patients have a better sense of control over their medication behavior. In addition, recurrent stroke was another influencing factor of medication adherence among stroke patients, consistent with the study by Wang et al. (21). This suggests that patients who have had two or more strokes have better drug compliance. This may be because the patients' clinical outcome becomes worse after a stroke recurrence, which increases their awareness and attention to the role of drugs in secondary prevention (21, 44). Similarly, patients with severe consequences may obtain more obvious benefits or relief from medication and are therefore more likely to receive prescription medication (21).

The foremost and novel finding was that patient activation is positively correlated with mediation adherence. That is, the higher the patient activation was, the better their medication adherence was. This expands the findings of others (23, 27, 45). A previous study has demonstrated that patients with chronic disease with the lowest levels of PAM (level 1) are >2.5 times more likely to self-report that they were missing 2 or more days of their medications in the past 7 days than patients with higher activation levels (level 4) (OR = 2.65; 95%: 1.74–4.03) (42). In another study, the PAM score was also positively correlated with self-reported medication adherence in people with chronic illness (OR = 1.18;95% CI:1.09, 1.29) (46). The reason may be that more self-management knowledge in active patients is associated with better medication compliance. Previous studies have also provided similar views (18). A cohort study (47) interviewed 130 stroke survivors and found that the current status of their disease knowledge was worrisome. For example, more than half of the patients could not tell the common risk factors and preventive behaviors for stroke. At the same time, their medication behavior was poor. For example, almost one-third of patients report noncompliance, which reflects the potential positive correlation between knowledge and medication compliance (47). Later, a qualitative study from England studied the barriers to medication adherence reported from the perspectives of stroke survivors, nurses, and doctors and reached similar conclusions (14, 16). According to reports, patients who do not know much about medications are more likely to have medication compliance problems such as self-discontinuation of the medications (48). In fact, due to the misleading traditional Chinese medicine culture, most people in China believe that “If it's medicine, it's toxic” (49). If stroke patients do not have scientific knowledge about drugs and secondary prevention, such as the benefits of drugs, they may worry excessively or even exaggerate the side effects of the drugs. This is a common misunderstanding from the perspective of Chinese patients (16). Skills refer to patient planning and organization skills (17), and this factor has a significant effect on medication compliance. Better skills and better medication compliance are still positively correlated (50). Skills related to medication mainly include (i) ability to reasonably analyze self-existing medication noncompliance problems and try to solve them; (ii) ability to make correct decisions about daily medication behavior, including how to correct missed medications; and (iii) ability to find and use various resources, such as telephone resources, internet resources, community health centers, and hospitals; (iv) ability to establish partnerships with healthcare professionals, accurately report the effects and side effects of drugs, discuss with medical experts, and properly choose treatment options; and (v) ability to take action (15, 51). Perhaps the most important thing is to develop and implement a short-term and specific action plan., i.e., within the time limit of 1 week, patients can aim to accurately take their medications, participate in physical exercise, and eat a healthy diet. In terms of beliefs, the need for more attention to medications and belief in the necessity of the medications are the two most common influencing factors. Studies have already indicated this. In a meta-analysis review, the impact of beliefs and concerns about the necessity of medication on the compliance of patients who need to take medication for a long time was evaluated, and the study finally showed that the enhancement of this belief improves the compliance of patients with medication. In addition, studies have also pointed out that interventions aimed at improving patients' perceived needs, necessities, and concerns about medication have improved their medication compliance (52, 53). The beliefs that pertain to positivity seem to play an important role in medication compliance. Therefore, medical staff should pay attention to the fact that it is important to build positive knowledge and beliefs in patients with lower scores (level 1 and level 2), and it is more necessary to maintain skills and self-confidence in patients with higher scores (level 3 and level 4). These results are closely related to clinical applications because they confirm the theoretical basis for medical staff to provide patients with knowledge, education, belief guidance, and recommended self-care skills. This can help patients achieve a higher level of enthusiasm so that they can continue to adhere to the doctor's medication when staying at home in the future. Based on our study, the guidelines for strategic positive action are as follows: For patients at activation level 1, healthcare personnel should strengthen patients' cognition of stroke, that is, inform the patients that stroke is a chronic disease that is preventable and controllable, and compliance with medicine is key. Healthcare personnel should also make patients clear about their responsibilities in the process of disease management and help them understand the importance of playing an active role in medication self-management (15). For patients at level 2, medical staff can train patients about the knowledge and skills associated with healthy self-care, encourage patients to start making small changes in healthy behaviors, and use the principles of simplicity, acceptance, and maintenance to gradually change the role of patients from passive to active. At the same time, medical staff can help patients build self-confidence through successfully changing behaviors that are easy to change and easy to persist. For patients at level 3, medical staff help patients develop plans, set dates to enhance behavior change, and give encouragement and praise when patients achieve progress or reach a goal. At the same time, patients should be encouraged to seek the support and help of family or friends to supervise and consolidate the established behavior changes. For patients at level 4, medical staff should gradually cultivate patients' ability to solve various self-care problems but encourage them to seek professional medical help in time when the disease condition is in crisis, teach patients to relieve stress, maintain patients' confidence, and maintain patients' trust in the medical staff. Finally, medical staff should enable patients to better implement behavioral changes in medical compliance and medication adherence.

### Limitations

This study has some limitations as follows. In terms of sample representativeness, the convenience sampling method was adopted in this study. This type of sampling cannot conclude the population (statistical generalization) (54). Only stroke patients who were neurology outpatients at one third-class hospital in Guangzhou, China, were enrolled. This may have caused selection bias. In addition, this study used intentional sampling and extrapolating the conclusion to other diseases also has some limitations. Additionally, the questionnaire used in this study suggested that patients take the past 1 month as their reference; therefore, there may have been a recall bias. Moreover, in the cross-sectional design of the questionnaire survey, social idealism is also considered to be a common and influential bias (55); patients surveyed may tend to choose the answers they think are expected by healthcare workers. Regarding the patients who refused to be investigated, it is not known whether their characteristics are different from those of the individuals who participated in this study. In addition, this study investigated the medication compliance of participants but was not able to obtain all clinical outcomes, such as some biochemical examinations, ultrasound examinations, and other objective data (data loss rate > 20%). Therefore, it was impossible to conduct an in-depth analysis. Lastly, the study was conducted 6 years ago, according to the Stroke Health Manager Training Project, which was carried out by the Chinese Government in 2017, and this study may not reflect the potential improvements after that program.

Future research can continue with attention to the following aspects: increase the sample size and expand the representativeness of the sample and improve the collection of objective data, such as biochemical examination, imaging examination, and other empirical data, in evaluating the difference in compliance with different medications. According to the practice guidelines for improving medication compliance and patient enthusiasm analyzed in this study, medical staff can intervene with stroke patients and discuss the medication compliance of patients after intervention in clinical practice.

## Conclusions

Medication nonadherence is a worrisome issue. Patients in Guangzhou, China, with ischemic stroke demonstrated low medication adherence. The reasons behind this are complex and diverse. A significant independent influence on medication compliance in patients with stroke was patient activation. Therefore, healthcare providers should pay attention to the activation level in patients with stroke, including their sense of self-care responsibility, confidence, knowledge, and skills for sustained medication adherence. In the future, it is hoped that targeted intervention measures can be developed to effectively improve the medication compliance of Chinese patients with stroke.

## Data Availability Statement

The raw data supporting the conclusions of this article will be made available by the authors, without undue reservation.

## Ethics Statement

Ethical approval was obtained from the School of Nursing, Sun Yat-sen University and The Third Affiliated Hospital of Sun Yat-sen University. The patients/participants provided their written informed consent to participate in this study.

## Author Contributions

WS and L-hW have made a substantial, direct, and intellectual contribution to the work and approved it for publication.

## Conflict of Interest

The author declares that the research was conducted in the absence of any commercial or financial relationships that could be construed as a potential conflict of interest.

## Publisher's Note

All claims expressed in this article are solely those of the authors and do not necessarily represent those of their affiliated organizations, or those of the publisher, the editors and the reviewers. Any product that may be evaluated in this article, or claim that may be made by its manufacturer, is not guaranteed or endorsed by the publisher.
